# Sociodemographic and lifestyle determinants of multimorbidity among community-dwelling older adults: findings from 346,760 SHARE participants

**DOI:** 10.1186/s12877-023-04128-1

**Published:** 2023-07-10

**Authors:** Wenqing Ni, Xueli Yuan, Yan Zhang, Hongmin Zhang, Yijing Zheng, Jian Xu

**Affiliations:** Department of Elderly Health Management, Shenzhen Center for Chronic Disease Control, No.2021, Buxin Rd, Shenzhen, Guangdong 518020 P.R. China

**Keywords:** Older adults, Multimorbidity, Associated factors

## Abstract

**Background:**

This study aimed to investigate the prevalence of multimorbidity and its associated factors among the older population in China to propose policy recommendations for the management of chronic diseases in older adults.

**Methods:**

This study was conducted based on the 2021 Shenzhen Healthy Ageing Research (SHARE), and involved analysis of 346,760 participants aged 65 or older. Multimorbidity is defined as the presence of two or more clinically diagnosed or non self-reported chronic diseases among the eight chronic diseases surveyed in an individual. The Logistic analysis was adopted to explore the potential associated factors of multimorbidity.

**Results:**

The prevalences of obesity, hypertension, diabetes, anemia, chronic kidney disease, hyperuricemia, dyslipidemia and fatty liver disease were 10.41%, 62.09%, 24.21%, 12.78%, 6.14%, 20.52%, 44.32%, and 33.25%, respectively. The prevalence of multimorbidity was 63.46%. The mean count of chronic diseases per participant was 2.14. Logistic regression indicated that gender, age, marriage status, lifestyle (smoking status, drinking status, and physical activity), and socioeconomic status (household registration, education level, payment method of medical expenses) were the common predictors of multimorbidity for older adults, among which, being women, married, or engaged in physical activity was found to be a relative determinant as a protective factor for multimorbidity after the other covariates were controlled.

**Conclusion:**

Multimorbidity is prevalent among older adults in Chinese. Guideline development, clinical management,and public intervention should target a group of diseases instead of a single condition.

## Introduction

The global population is aging rapidly due to a combination of increased life expectancy and falling fertility. China is also facing many serious challenges related to aging. As one of the fastest-aging countries globally, China had 190.64 million people aged 65 or older in 2020, accounting for 13.5% of the total population [1]. There will be 400 million Chinese citizens aged 65 or older by 2030 [2]. With an aging population, the number of people with multimorbidity (defined as the coexistence of two or more chronic diseases in the same person) is expected to rise at a rate of more than 1% per year by 2030 as the population ages [3, 4]. Multimorbidity is associated with impaired functional ability, higher healthcare costs, poorer quality of life and an increased mortality risk, challenging healthcare systems around the world [5, 6]. The challenges require a radical shift from disease-specific research to a more holistic view of our health. Therefore, studies focused on multimorbidity are being increasingly emphasized [7].

Previous studies on the prevalence of multimorbidity and its patterns may not be comparable due to large heterogeneity in the study samples, definitions, eligible diseases, and analytical methods [8, 9]. Asogwa and co-researchers found that the prevalence of multimorbidity was 26.2% for sub-Saharan Africa, 29.5% for Asia, 31.8% for East Asia, 33.1% for the Middle-East and North Africa, 44% for Europe and Central Asia and 50.4%% for Latin America and the Caribbean [8]. Two recent nationally representative studies indicated that the prevalence of multimorbidity ranged from 42.4% to 56.73% among middle-aged and older adults in China [10–12]. However, so far, the studies on multimorbidity used the disease self-reporting measures predominantly, which could be affected by recall bias [8, 10–12]. In addition, previous studies investigating multimorbidity were mostly conducted in a small rural area in China with a limited sample size [13, 14]. It is therefore necessary to accurately identify the prevalence of multimorbidity using more objective clinical diagnoses without recall bias among older Chinese adults, which is important for providing essential information for public intervention and clinical management.

Arokiasamy and co-researchers found that women had significantly higher risk of multimorbidity compared with men, while other researchers showed a non-significant correlation [15, 16]. A review revealed that most studies reported a higher risk of multimorbidity with lower education, while some studies reported a lower risk with lower education [8]. Studies have found opposite results on the relationship between multimorbidity and income [15, 17]. A better understanding of the factors impacting the prevalence of multimorbidity is critical to develop appropriate guidelines for the management of multimorbidity, generate new hypotheses on etiology underlying associations, facilitate studies to identify risk factors, and identify groups of people to target for screening interventions. Therefore, this study sought to accurately quantify the prevalence of multimorbidity among older adults in Shenzhen, a financial center in China, and then analyze the potential factors associated with multimorbidity.

## Methods

### Study population

The Shenzhen Healthy Ageing Research (SHARE) is a community-based prospective cohort study of non-communicable diseases (NCDs) among older adults. The research was designed to study the etiology and prognosis of NCDs. We established SHARE in Shenzhen based on the older adult health management project of the National Basic Public Health Service. The older adults health management project recruited people aged 65 and older from the lists of all residents registered at local community health centres in Shenzhen. Recruitment activities included pasting posters or placing foldings in local community health centers and other public places. Electronic posters or messages were also distributed through all the open WeChat groups of local community health centres’ staff, to make the survey available to the close contacts easily. Older adults also were identified by general medical practitioner and referred to nurses or identified by nurses based on review of medical records and then were directly recruited. Moreover, the staff of the local community health centers recruited the older adults in their service communities to participate in the survey by telephone. The participants in SHARE comprised all older adults attended to the older adult health management project of the National Basic Public Health Service in Shenzhen was started in 2018. Since 2018, new recruitment or follow-up surveys have taken place almost every year, with each survey on new-recruited older adults taking twelve months to complete. In this cross-sectional study, the fourth-year participants of SHARE (2021) were selected, and 352,022 participants were enrolled. The participants enrolled accounted for 62.28% (352, 022/565,217) of the resident population of older adults in Shenzhen based on the data from the 2020 population census. Participants excluded from the study, 5,262, were those who did not complete the questionnaires or provide a fasting blood samples, or were unable to attend physical examinations or abdomens B-ultrasound tests. Finally, 346,760 participants were included in the final data analysis. All procedures described in this study were performed in accordance with the principles of the Declaration of Helsinki. Our study was approved by the Shenzhen Center for Chronic Disease Control Human Ethics Committee (No. SZCCC-2021-061-01-PJ). As claims data were provided anonymously, informed consent was exempted by the Shenzhen Center for Chronic Disease Control Human Ethics Committee.

### Data collection

Older adults enrolled in SHARE received face-to-face questionnaires, physical examinations, electrocardiography measurement, laboratory biochemistry tests, abdomens B-ultrasound tests and etc by trained staff of local community health service centers [18–20]. Data was collected in medical examination rooms at local community health centers in the older adult residential areas. The full details of the survey procedures, measureing methods and baseline variables have been described in previous publications [18–20]. In brief, all participants completed a standardized questionnaire including sociodemographic status, lifestyle, medical history, family health history, genetic history, medication use, hospitalization history, immunization history, etc [18]. A detailed physical examination was performed to collect information on systolic blood pressure (SBP), diastolic blood pressure (DBP), weight, height, etc [18]. Body mass index (BMI) was calculated by dividing body weight (in kilograms) by the square of height (in m). The fasting blood sample of study participant was collected to obtain information on total cholesterol(TC), triglyceride(TG), high-density lipoprotein cholesterol (HDL-C), low-density lipoprotein cholesterol (LDL-C), fasting plasma glucose (FBG), haemoglobin (Hb), uric acid (since August 1, 2020), creatinine, etc [19]. Serum creatinine was used to calculate the estimated glomerular filtration rate (eGFR) using the Modification of Diet in Renal Disease equation for Chinese [21]. The color Doppler ultrasound scan of the abdomens was performed to determine the presence of fatty liver disease [20].

### Multimorbidity and chronic diseases

Multimorbidity was defined as reported coexistence of two or more chronic diseases within one person. For this study, the following eight chronic diseases were selected based on the most frequently mentioned diseases for multimorbidity measurements by previous literatures, which were considered to significantly impact long-term treatment and decrease functional performance and quality of life among the older Chinese population: hypertension, diabetes, dyslipidemia, hyperuricemia, chronic kidney disease (CKD), fatty liver disease, anemia, and obesity [11, 12]. Additionally, the inclusion criteria also considered a core list of chronic conditions for any multimorbidity measurement as recommended by a systematic review [22]. Although it was controversial whether obesity should be considered as a chronic condition or a risk factor in multimorbidity studies, the British Academy of Medical Sciences recommended that obesity should be reported in multimorbidity research wherever possible [23]. All diseases and conditions were defined as binary variables (present vs. absent). The definition of hypertension, diabetes, dyslipidemia, CKD, anemia and obesity have been described in our previous studies [18, 19]. Hyperuricemia was defined as a serum uric acid level > 420 umol/L [24]. The typical manifestations of fatty liver disease were enlargement of liver and blunt edge Angle [20, 25]. The near field echo of liver was diffusely enhanced, higher than that of spleen and kidney, while the far field echo was weakened. Hepatic duct structure was not clear [20, 25].

### Covariates

Sociodemographic variables included age (65–69, 70–74, 75–79,≥80), gender (men, women), household registration(yes, no), nationality(Han nationality, other), education level (illiterate, primary education, junior school education, senior school education, senior school education above) and marriage status (married, widowed, divorced, single), payment method of medical expenses(urban employee basic medical insurance (UEBMI), urban resident basic medical insurance (URBMI), new rural cooperative medical scheme (NRCMS), other, Out-of-pocket medical expenses (OOPME)). In this study, we define the term “moderate to vigorous physical activity” to refer to at least some sweating and shortness of breath caused by engaging in physical activity, and the term “light physical activity” to refer to no sweating or shortness of breath caused by engaging in physical activity. In addition, moderate to vigorous physical activity at least once a week was classified as “yes” for physical activity status [18, 26]. Current smokers were those who reported smoking tobacco products at survey. Former smokers were those who had habitually used tobacco products in the past but not so currently for at least one month. Never smokers were those who reported having never smoked tobacco throughout their life [27]. Alcohol consumption status was categorized into three groups of never drinker (almost never), current drinker (once per day or more to once per month drunk during the past year) and former drinker (quitted drinking in the past year) [26].

### Statistical analyses

Descriptive statistics were expressed as mean and SD for quantitative variables and as frequencies and proportions for categorical variables. Chi-square tests were used for categorical variables in the univariate analysis. Logistic regression analysis was performed to explore the association between the prevalence of multimorbidity and associated risk factors. In the multivariate logistic regression model, the prevalence of multimorbidity was defined as the dependent variable, and age, sex, household registration, nationality, education level, marriage status, payment method of medical expenses, smoking status, drinking status, and physical activity status were defined as the independent variables. All statistical analyses were performed using the SAS software package (version 9.4, SAS Institute, Inc. Cary, NC, USA). All differences were found to be statistically significant using two-tailed significance tests (*P* < 0.05).

## Results

### Sociodemographic and lifestyle characteristics of participants

The sociodemographic characteristics of the included participants are described in Table 1. A total of 346,760 participants were included in this study, in which nearly 45.21% were men and 54.79% were women, with the average age being 71.43 ± 5.30. Among the participants, 69.00% were non-household registration residents, 99.49% of the participants were Han nationality, 96.24% were married, 26.93% had attained a junior school education, 9.34% were enrolled in UEBMI, 10.95% were current smokers, 78.48% were engaged in physical activity, 13.58% were current drinkers.

**Table 1:** Sociodemographic and lifestyle characteristics of participants

Characteristics	General (N = 346,760)
Age(year)	71.43 ± 5.30
Gender, N(%)	
Men	156,753 (45.21)
Women	190,007 (54.79)
Household registration, N(%)	
No	239,270 (69.00)
Yes	107,490 (31.00)
Nationality, N(%)	
Han	344,991 (99.49)
Others	1769 (0.51)
Marriage status, N(%)	
Divorced/ Widowed/ Single	13,028 (3.76)
Married	333,732 (96.24)
Education, N(%)	
Illiterate	22,782 (6.57)
Primary education	124,214 (35.82)
Junior school education	93,377 (26.93)
Senior school education	43,407 (12.52)
Senior school education above	62,980 (18.16)
Payment method of medical expenses, N(%)	
OOPME	265,360 (76.52)
UEBMI	32,398 (9.34)
URBMI	45,766 (13.20)
NRCMS	1934 (0.56)
Others	1302 (0.38)
Smoker status, N(%)	
Never smoker	272,737 (78.65)
Former smoker	36,055 (10.40)
Current smoker	37,968 (10.95)
Drinker status, N(%)	
Never drinker	285,153 (82.23)
Former drinker	14,521 (4.19)
Current drinker	47,086 (13.58)
Physical activity, N(%)	
No	74,625 (21.52)
Yes	272,135 (78.48)

### Prevalence of chronic diseases

The mean BMI, SBP, DBP, FBG, Hb, eGFR, uric acid, TC, TG, LDL-C and HDL-C values among all the 346,760 participants were 24.04 ± 3.19 Kg/m^2^, 137.26 ± 17.35 mm Hg, 78.47 ± 10.52 mm Hg, 5.93 ± 1.62 mmol/L, 13.78 ± 1.54 g/dL, 96.58 ± 26.82 mL/min/1.73m^2^, 349.70 ± 107.41umol/L, 5.09 ± 1.16 mmol/L, 1.55 ± 1.02 mmol/L, 3.01 ± 0.94 mmol/L and 1.37 ± 0.41 mmol/L, respectively (data not shown). The prevalences of obesity, hypertension, diabetes, anemia, CKD, hyperuricemia, dyslipidemia and fatty liver disease were shown in Table 2.

**Table 2:** Prevalence of chronic diseases

Characteristics	Prevalence(N = 346,760)
Obesity	
No	310,656 (89.59)
Yes	36,104 (10.41)
Hypertension	
No	131,469 (37.91)
Yes	215,291 (62.09)
Diabetes	
No	262,794 (75.79)
Yes	83,966 (24.21)
Anemia	
No	302,441 (87.22)
Yes	44,319 (12.78)
CKD	
No	325,476 (93.86)
Yes	21,284 (6.14)
Hyperuricemia	
No	275,595 (79.48)
Yes	71,165 (20.52)
Dislipidemia	
No	193,067 (55.68)
Yes	153,693 (44.32)
Fatty live disease	
No	231,477 (66.75)
Yes	115,283 (33.25)

### Prevalence of multimorbidity

**Fig. 1:**
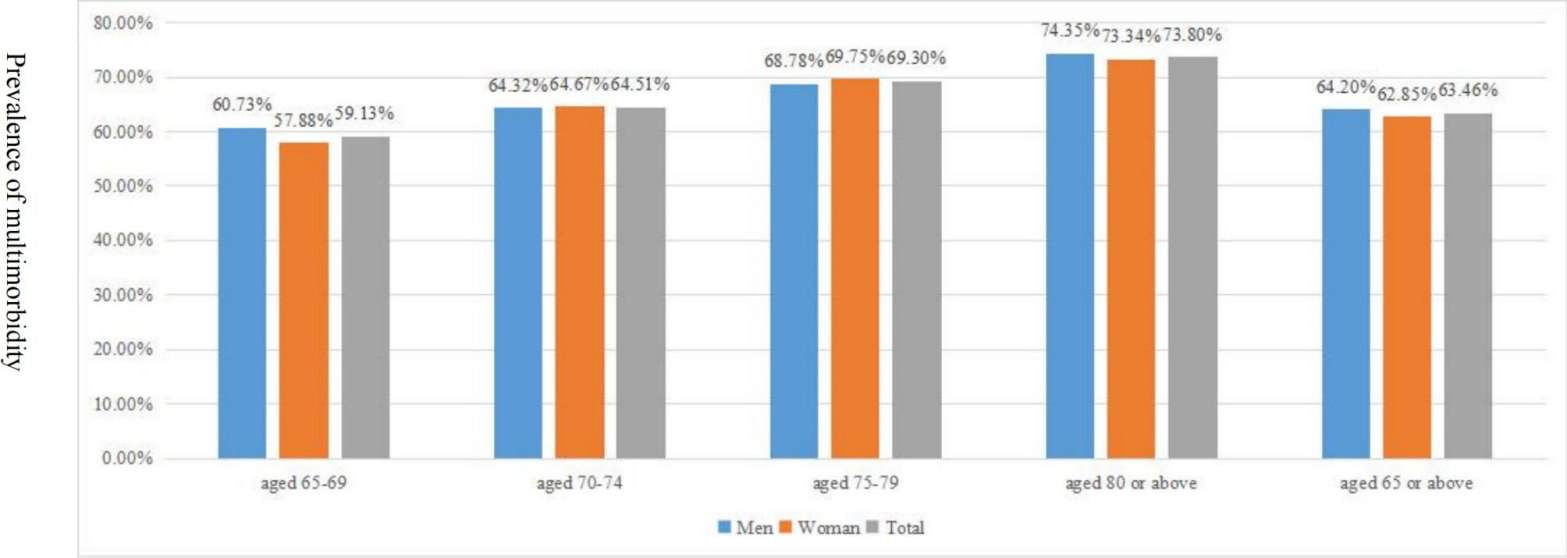
Prevalence of multimorbidity by age and gender

**Fig. 2:**
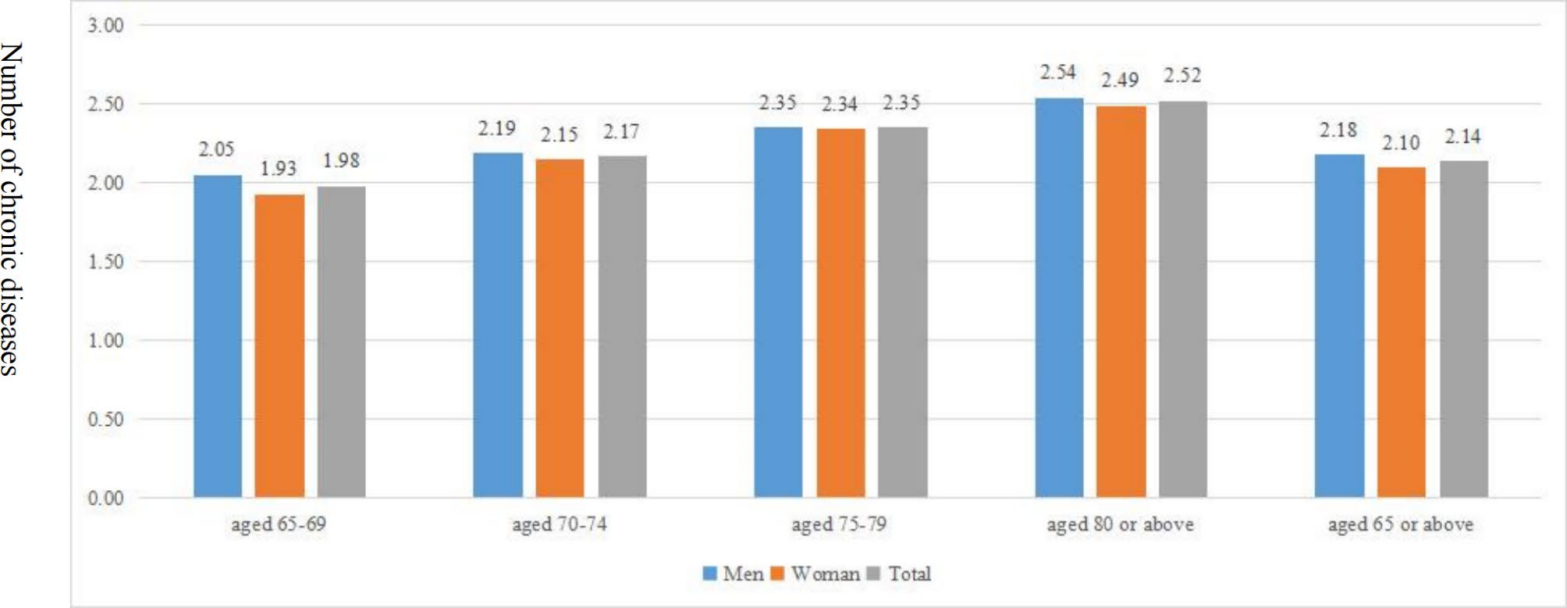
Number of chronic diseases by age and gender

Figure 1 displays the prevalence of multimorbidity by age and gender. The prevalence of multimorbidity was 63.46%, specifically, 64.20% in older men and 62.85% in older women, respectively. The mean count of chronic diseases per participant was 2.14. The mean count of chronic diseases per men and women participant was 2.18 and 2.10, respectively (as shown in Fig. 2). Multimorbidity was significantly more prevalent or the mean count of chronic diseases higher in men than in women and generally significantly increased with age (as shown in Figs. 1 and 2). The prevalence of multimorbidity was lower in participants with non-household registration and higher in those who attended URBMI or were divorced/ widowed/ single (as shown in Table 3). The prevalence was higher among participants with at least senior school education above, and lower in current smokers and current drinkers compared with their counterparts (as shown in Table 3).

**Table 3:** Prevalence of multimorbidity in different subgroup

Characteristics	No of Multimorbidity	Prevalence of Multimorbidity (%)	χ^2^ value	*P* value
Household registration			3500.582	<0.001
No	144,083	60.22		
Yes	75,973	70.68		
Nationality			4.244	0.039
Han	218,975	63.47		
Others	1081	61.11		
Marriage status			103.044	<0.001
Divorced/ Widowed/ Single	8815	67.66		
Married	211,241	63.30		
Education			826.202	<0.001
Illiterate	14,052	61.68		
Primary education	76,256	61.39		
Junior school education	58,831	63.00		
Senior school education	28,212	64.99		
Senior school education above	42,705	67.81		
Payment method of medical expenses			3511.07	<0.001
OOPME	161,459	60.85		
UEBMI	23,376	72.15		
URBMI	33,216	72.58		
NRCMS	1126	58.22		
Others	879	67.51		
Smoker status			619.379	<0.001
Never smoker	172,940	63.41		
Former smoker	24,577	68.17		
Current smoker	22,539	59.36		
Drinker status			181.793	<0.001
Never drinker	181,033	63.49		
Former drinker	9881	68.05		
Current drinker	29,142	61.89		
Physical activity				
No	47,173	63.21	2.506	0.113
Yes	172,883	63.53		

### Factors associated with multimorbidity

Binary logistic regression analysis was carried out with the presence or absence of multimorbidity as the dependent variable, and factors in univariate analysis as independent variables to determine the factors influencing multimorbidity. Table 4 presents the results of the factors associated with multimorbidity, reporting the adjusted *OR* (*AOR*) and 95% *CI.* Compared with participants belonging to the relatively healthy class, those classified into the multimorbidity class were men, most likely to be single/divorced/widowed. Those who were household registration were 1.210 times more likely to have multimorbidity. Participants with higher education (junior school education and above) were associated with higher probability of being in the multimorbidity class. Compared to OOPEM, all types of non-OOPME (except NRCMS) were associated with an increased likelihood of being in the multimorbidity class, with the highest AOR for URBMI. Regarding smoking status, respondents who were former smoker had a higher possibility of being in the multimorbidity class, while who were current smoker had a lower possibility of being in the multimorbidity class. Similarly, respondents who were former drinker had a higher possibility of being in the multimorbidity class, while who were current drinker had a lower possibility of being in the multimorbidity class. Compared with those in the 65–69 age group, participantsaged 70 or older were more likely to have multimorbidity.

**Table 4:** Multivariable logistic analysis of the factors associated with multimorbidity

Characteristics	*AOR*(95%*CI*)	*P* value
Gender		
Men	Reference	
Women	0.982 (0.965-1.000)	0.047
Household registration		
No	Reference	
Yes	1.210 (1.185–1.236)	< 0.001
Nationality		
Others	Reference	
Han	0.999 (0.907-1.100)	0.982
Marriage status		
Divorced/ Widowed/ Single	Reference	
Married	0.925 (0.891–0.962)	< 0.001
Education		
Illiterate	Reference	
Primary education	1.020 (0.990–1.051)	0.191
Junior school education	1.062 (1.030–1.095)	< 0.001
Senior school education	1.103 (1.065–1.142)	< 0.001
Senior school education above	1.096 (1.060–1.134)	< 0.001
Payment method of medical expenses		
OOPME	Reference	
UEBMI	1.377 (1.337–1.419)	< 0.001
URBMI	1.407 (1.370–1.445)	< 0.001
NRCMS	0.920 (0.840–1.008)	0.073
Others	1.171 (1.041–1.317)	0.008
Smoker status		
Never smoker	Reference	
Former smoker	1.218 (1.185–1.253)	< 0.001
Current smoker	0.884 (0.862–0.907)	< 0.001
Drinker status		
Never drinker	Reference	
Former drinker	1.181 (1.136–1.228)	< 0.001
Current drinker	0.956 (0.934–0.978)	< 0.001
Physical activity		
No	Reference	
Yes	0.961 (0.944–0.977)	< 0.001
Age group		
65–69	Reference	
70–74	1.221 (1.201–1.241)	< 0.001
75–79	1.466 (1.433-1.500)	< 0.001
≥ 80	1.756 (1.709–1.805)	< 0.001

## Discussion

To the best of our knowledge, this study was one of the few to explore multimorbidity prevalence based on non self-reported disease data among a large older Chinese population. About 63.46% of all participants had multmorbidity. It is evident that chronic and multi-morbidity is a prevailing problem among older adults in China.We found that participants who were men, older, single/divorced/widowed, higher socioeconomic status (including household registration residence, with higher education and medical insurance), former smoker, or former drinker were more likely to have multimorbidity. Physical activity was found to decrease the probability of multimorbidity.

In our study, the prevalence of multimorbidity was 63.46% among the older Chinese population, which differed from the prevalence reported in previous studies in China and other low-middle and high income regions [3, 12, 28].The previous studies on the prevalence of multimorbidity may not be comparable due to differences in the sampling methods, sampling size, and number of chronic diseases enrolled.

Consistent with the previous studies, the prevalence of cardiometabolic multimorbidity in men was slightly higher than that in women [29]. The positive association of multimorbidity with age was consistent with a study comparing 27 low-income and middle-income countries and 1 high-income countries using the World Health Survey, and other reviews on multimorbidity in South Asia and low-income and middle-income countries as well as reviews from high-income countries [30–34]. At the family and social network layer, being divorced/widowed/single was a risk factors for multimorbidity. It was consistent with previous studies, which indicated that support from a spouse was the most direct social support older adults could receive, while divorced/widowed/single older adults who lived with a smaller social network, were more likely to have multimorbidity [35].

Lifestyle variables were found to be influencing factors for multimorbidity. Older adults engaged physical activity were less likely to suffer from multimorbidity. Previous studies showed that there was an inverse association between physical activity and multimorbidity among adults aged 65 or older, and this association was easier to be found in studies that include more than ten chronic diseases [36, 37]. The reason is simple: while not a disease itself, the behavior of physical inactivity is by far the risk factor for many chronic diseases. In addition, participants engaged in physical activity may generally pay more attention to their health. Unsurprisingly, therefore, the group with the highest level of physical inactivity had a higher likelihood of having multimorbidity.

We found a higher prevalence of multimorbidity in former smokers than in current smokers and never smokers. With the sociodemographic characteristics controlled, older adults who were former smokers reported higher levels of fair or poor health, COPD, and four or more chronic conditions compared with those who were never smokers and similar levels of fair or poor health, four or more chronic conditions, and limitations in social participation compared with those who were current smokers [38]. Similarly, compared to respondents who were never drinkers and current drinkers, former drinker were more likely to have multimorbidity. Compared to lifetime abstainers, former alcohol consumption was associated with more depressive symptoms, and worse global quality of life and social functioning, while current drinking was associated with less anxiety, depression and better health-related quality of life [39]. The previous study focused on older adults found that former alcohol users had more ER/ED visits and current moderate alcohol users at all levels had fewer ER/ED visits than lifetime abstainers [40]. The more detailed measurement of alcohol consumption and smoking is necessary in future research. Because of our cross-sectional study design, we could not make causal inferences between drinking, smoking and multimorbidity.

In contrast to previous studies in developed countries showing that people with lower socioeconomic status were more likely to have multimorbidity related to cardiometabolic conditions, our study found that respondents with a higher socioeconomic status were more likely to be in the multimorbidity class, including higher education, household registration residence and medical insurance [41, 42]. Similarly, previous studies documented a significantly higher probability of the vascular multimorbidity class for those with higher socioeconomic status among middle-aged and older adults in China [3, 10, 43]. In a review of 39 studies on multimorbidity focused on low-income and middle-income, Asogwa et al. showed that higher income and urban residents were associated with higher prevalence of multimorbidity of NCDs [8]. Moreover, other studies found that urban residents in China were more likely to belong to multimorbidity groups [11, 44]. This can be explained by the fact that respondents with a higher socioeconomic status have a higher possibility of avoiding physically demanding tasks and consume high-calorie foods or more seafood, which may lead to a high possibility of chronic diseases such as hypertension, obesity, fatty liver disease, dyslipidemia, hyperuricemia, and diabetes. Moreover, China has achieved almost universal coverage of social health insurance through three major schemes: UEBMI for urban employees, URBMI for urban residents and NRCMS for rural residents [45]. In most regions, NRCMS funds are pooled at the county level, while URBMI and UEBMI are pooled at the municipal (prefecture) level, implying that there are thousands of health insurance pools in China [45]. In addition, China has still maintained its unique household registration system, also known as “Hukou” originally designed for limiting population mobility [46]. Those who work or live outside of their household registration location are referred to as internal migrants. Previous studies showed that 80% of internal migrants paid entirely out-of-pocket for their recent medical consultations [47]. Shenzhen is an immigrant city, and the non-registered older residents are mainly internal immigrants. Compared to household registration older adults, non-registration older adults take more labor-intensive and lower-paid jobs such as cleaning, take care of children, and catering. In our study, being a non-registered older resident was a protective factor of multimorbidity. It could be a consequence of the so-called “healthy migration effect” [48]. Numerous studies also showed how the migrant population had a high level of health in the host countries, even higher than the host population [48].

The main strength of this study was the large sample size with non self-reported disease data to illustrate multimorbidity prevalence among the older Chinese population. The multimorbidity was measureed by clinical diagnosis, which could help accurately identify the prevalence of multimorbidity among the older Chinese population. This study also had several limitations. One is that this study was conducted only in one city of China, and the generalizability of the findings to other cities in China and other countries may be limited. In addition, the cross-sectional design of the study made it impossible to draw confident causal conclusions.

## Conclusion

Multimorbidity is prevalent among older Chinese adults. Multimorbidity is prevalent and disproportionally distributed across varying personal innate, marriage status, lifestyle behavioral characteristics, and socioeconomic characteristics among older adults. It is important to shift from single disease-oriented clinical guidelines to multimorbidity frameworks in older Chinese populations. Screening of high-risk populations and integrated management of multimorbidity should be top priorities in Shenzhenand listed as important public health intervention measures for implementation.

## Data Availability

The datasets used and/or analysed during the current study will be available from the corresponding author on reasonable request.

## Abbreviations

BMI: Body mass index
CKD: Chronic kidney disease
DBP: Diastolic blood pressure
eGFR: estimated glomerular filtration rate
FBG: Fasting plasma glucose
Hb: Haemoglobin
HDL-C: High-density lipoprotein cholesterol
LDL-C: Low-density lipoprotein cholesterol
NCDs: Non-communicable disease (NCDs)
NRCMS: New rural cooperative medical scheme
OOPME: Out-of-pocket medical expenses
SHARE: Shenzhen Healthy Ageing Research
SBP: Systolic blood pressure
TC: Total cholesterol
TG: Triglyceride
UEBMI: Urban employee basic medical insurance
URBMI: Urban resident basic medical insurance
